# How urban residential land transfer policies affect the housing market—evidence from Beijing, China

**DOI:** 10.1371/journal.pone.0334886

**Published:** 2025-10-22

**Authors:** Chunyan Li, Deqi Wang, Wei Liang, Fei Zhang

**Affiliations:** 1 School of Urban Economics and Public Administration, Capital University of Economics and Business, Beijing, China; 2 School of Statistics, Shanxi University of Finance and Economics, Shanxi, China; The Chinese University of Hong Kong, HONG KONG

## Abstract

The stable and healthy development of the residential market has always been one of the important tasks of the Government, and is of great significance to the maintenance of social stability and the well-being of residents. This paper utilizes the VAR model, the housing filtering model and the four-quadrant theoretical model to explore the comprehensive impact and mechanism of the land supply system on China’s housing market from the logical framework and institutional environment of land and housing. The results of the study show that the price of residential land supply has a positive effect on house prices, while the quantity of residential land supply has a negative effect on house prices; The land transfer patterns of “Restricted Land Price, on-site Lottery” and “Restricted Land Price, Restricted Selling Price and Compete for Quality” are conducive to the healthy development of the real estate market, but an excessive supply of land for leasing may push up the prices of commercial properties. Finally, in combination with China’s policy objective of “stabilizing land prices, housing prices and expectations”, it puts forward a number of policy recommendations to rationally control and guide the healthy development of real estate.

## 1. Introduction

Since 1998, with the reform of China’s housing welfare allocation system, it has prompted the rapid development of the Real Estate Industry. The prosperity of the real estate market has not only led to the development of industries such as finance and manufacturing, but has also led to employment in large numbers, which plays an important role in the development of the social economy [1]. China’s real estate development investment grew from 361.423 billion yuan in 1998 to 110,913 billion yuan in 2023, with an increase of 30.69 times. At the same time, the issue of the continuous rise in housing prices has become more and more visible [2]. According to the China Statistical Yearbook, the sales price of commercial housing in Beijing has risen from RMB 4,737 per square meter in 2003 to RMB 37,595 per square meter in 2023, which is an increase of nearly eight times in 20 years, and the increase in housing prices has exceeded the purchasing power of the public. In this situation, the Chinese government is focusing on solving the housing problem from the supply side. Land is the material basis for the survival and development of human society, and the high-quality development of the economy and the stable development of the real estate market cannot be separated from the support of land elements [3]. Supply-side structural reform of the land market helps to effectively integrate land, capital and labor factors, increase the effective supply of land, enhance total factor productivity, achieve economic transformation and development, and build a long-term mechanism for the healthy development of the real estate market [4]. Research on the residential housing market has primarily been conducted by scholars from the following two perspectives.

### 1.1 Land supply

The relationship between land supply and housing prices is a key focus of research in the real estate market. Existing studies have mainly focused on the impact of land supply prices, supply quantities, and supply patterns on the housing market. It is generally believed that the stronger the restrictions on land supply, the higher the housing prices [5]. From the perspective of land supply prices, scholars generally believe that land prices have a lag effect on housing prices. In other words, changes in housing prices will lead to changes in land prices in the short term, but in the long run, increases in land prices will lead to delayed increases in housing prices [6,7]. Research on land supply has shown that scholars have found that insufficient housing affordability is mainly due to limited land supply, and increasing the supply of land can effectively alleviate the pressure of rising housing prices [8,9]. However, some scholars disagree, arguing that residents are more sensitive to changes in the housing market, which in turn affects the supply of land [10,11]. When housing prices are high, local governments provide more residential land supply to maximize the revenue from residential land [12]. Different countries have different land supply patterns, which affect the real estate market in different ways. Gautier and Vuuren (2019) found that landlords on leased land in Amsterdam have to pay rent, while those on private land do not, resulting in higher house prices on private land than on leased land [13]. Land in China is publicly owned, and the main pattern of land allocation is through “Bidding, Auction and Listing”. Kuang and Li (2012) analyzed the real estate markets of 35 large and medium-sized cities in China and found that land allocation and tendering had a significant positive impact on housing prices, while land auctions had a significant negative impact on housing prices, and land listing had no impact on housing prices [14]. There are few studies on the impact of land supply structure on housing prices. Most current studies measure land supply structure using the proportion of commercial and residential land in the total land supply area [15], while some scholars use a comprehensive measure of land supply structure based on the proportions of residential land, industrial land, and infrastructure land supply [16]. This method of measuring land supply structure only indicates that a lower proportion of residential land supply leads to higher housing prices. It does not further subdivide residential land into property rights-type land and rental-type land to clarify the impact of residential land supply structure on housing prices, which is not conducive to a comprehensive understanding of the relationship between residential land supply structure and housing prices. Therefore, based on previous research, this paper further subdivides the residential land supply structure into rental residential land and property-type residential land. Among these, property-type residential land is further subdivided into the second-hand housing market, the ordinary commercial housing market, and the high-end commercial housing market. Using the four-quadrant theory model, this paper further analyzes the impact of the residential land supply structure on the real estate market from a theoretical perspective.

### 1.2 Macroeconomic policy

At the policy level, scholars have focused their research on the impact of fiscal policy [17], monetary policy [18], land policy [19] and real estate macro-control policies on the real estate market. In the research on real estate macro-policy regulation, the main focus is on the loan-to-value (LTV) ratio [20,21], stamp duty on property transactions [22], bank interest rates on housing loans [23], property tax [24], and other housing purchase restriction measures. Most studies have shown that real estate macro-policy moderation has been able to stabilize the growth of housing credit, thus slowing down the growth of transaction activity and reducing the growth rate of house prices [25,26]. However, in some regions the impact of these macro-control policies on the real estate market is not significant. Du and Zhang (2015) used counterfactual analysis to study the effects of China’s real estate purchase restriction policy and property tax on house prices, and found that such regulatory policies were able to dampen house prices in Beijing and Chongqing, but had no effect on house prices in Shanghai [27].

Unlike the existing literature, this paper adopts the change in China’s land transfer policy to study the impact of house prices. Since 1987, China’s land transfer system has gradually shifted from a single gratuitous allocation to a compensated allocation [28]. In the context of compensated land use, China further monetized housing allocation and began the exploration of the housing market [29]. Many literatures discuss the impact of macroeconomic policies on the real estate market, neglecting to view the logic behind house price growth from the perspective of local governments’ land supply strategies. The reset effect of land elements and the reform of the system are important sources of power to promote China’s rapid economic growth and build a long-term mechanism for the healthy development of the real estate market. According to Wind database statistics, the ratio of the area of industrial land and commercial residential land offered for sale and sold in Beijing in 2023 was 1.83:1, while the average price of land transaction for industrial land was only 26.05% of commercial residential land. What is the far-reaching impact of this “two-track” land supply model on China’s real estate market? With regard to the above questions, an interesting economic phenomenon can also be observed by observing the trends in land, housing and urban economic growth: cities and regions with faster economic growth in China also have relatively higher real estate prices. In other words, the growth of house prices and the growth of the urban economy are highly spatially overlapping. In order to better understand the policy logic behind this economic phenomenon, we choose Beijing, a megacity, as the object of our study, and examine it from the perspective of the local government’s land supply strategy. Two main questions are elaborated. What is the role of land transfer policies in cities with high economic growth? And how does land supply policy affect house prices? However, existing studies have not yet tackled the question of how changes in land transfer policies affect the housing market.

In order to answer these two questions, this paper intends to explore the comprehensive impact and mechanism of the land supply system on China’s residential housing market and from the logical framework and institutional environment of land and housing. The land transfer policy is divided into four aspects: land supply quantity, supply structure, supply patterns and supply price to analyze its impact on the housing market. In terms of supply quantity and supply price, this paper explores the impact of land supply quantity and supply price on house prices by constructing a VAR model for Granger causality test; In the study of supply patterns, the housing filter model and the four-quadrant theoretical model are used to analyze the five land transfer patterns: “Bidding, Auction and Listing”, “Restricted Land Price, Competitive Construction”, “Restricted house price, bid for land price”, “Restricted Land Price, on-site Lottery”, “Restricted Land Price, restricted selling price, and competing for quality”; With regard to the structure of land supply, a filtering model for the primary, secondary, tertiary and quaternary markets will be established to study the impact on the residential housing market of differences in the proportions of the structure of land supply for the rental housing, second-hand housing, general commercial housing and high-quality commercial housing markets.

The contribution of this study has three main aspects. Firstly, the logic behind house price growth is examined from the perspective of the local government’s land supply strategy, and explored the impact of the quantity, price, structure, and patterns of land transfers on the residential housing market. Second, a Vector Autoregressive Model (VAR) was established using quarterly data to explain the extent to which the quantity of residential land supply and the price of residential land supply affect changes in the residential real estate market. Thirdly, the residential filtering model and the four-quadrant theoretical model are employed to analyze the impact of the structure and the way of residential land transfer on the residential market of different grades, so that the depth and breadth of the research could be strengthened.

The structure of this paper is organized as follows: Section 2 is a factual analysis on the characteristics of China’s residential land transfer policy; Section 3 is the data source and research methodology; Section 4 is the analysis of the empirical results; Section 5 is the conclusion of this paper; Section 6 is Discussion.

## 2. A Factual analysis of the characteristics of China’s residential land transfer policy

### 2.1. The quantity of residential land supply shows a downward trend

**Fig 1:**
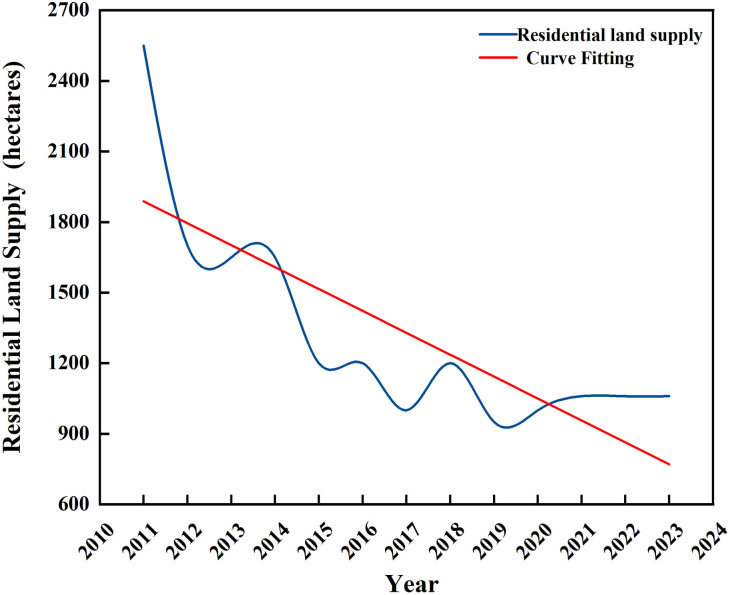
Trend of Beijing’s Residential Land Supply Program from 2011 to 2023. Data source: Beijing’s annual state-owned construction land supply plan.

As can be seen from Fig 1, the fluctuation of residential land supply quantity in Beijing from 2011–2023 is relatively high, and the general trend of the supply quantity shows a downward trend. Especially in the period 2011–2015, the quantity was reduced by nearly double, and the supply quantity dropped from the original 2,550 hectares to 1,200 hectares. After 2019, the supply of residential land has largely remained at a more stable level.

**Fig 2:**
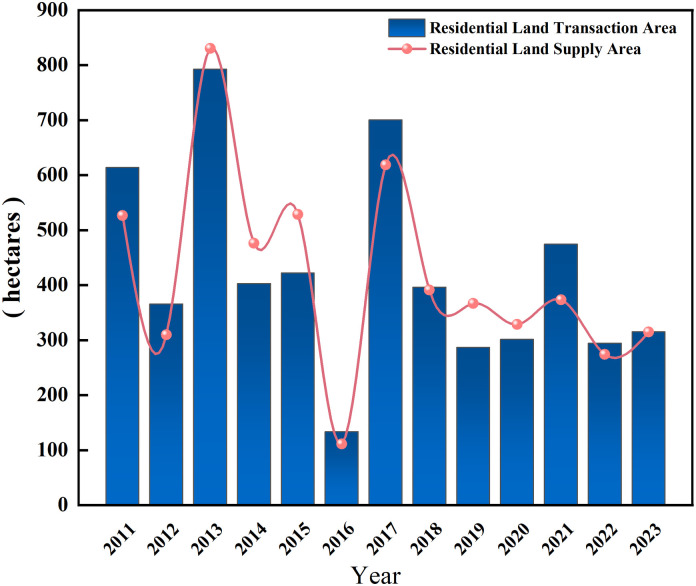
Trend of Residential Land Supply and Transaction Area in Beijing from 2011–2023. Data source: Wind database.

As can be seen from Fig 2, the number of residential land transactions is basically lower than the number of land supply during the period 2010–2021, which indicates that real estate developers are not highly motivated to acquire land. However, the quantity of residential land transactions was higher than the quantity of land supply in 2014 and 2015. Due to the wave of de-inventorying in the property market in many places in 2014, the land market picked up and the quantity of land transactions increased. After 2016, the Beijing municipal government has introduced a series of measures to restrict purchases and loans, and tightened the regulation of commercial residential properties. Under the tone of “housing without speculation” and “resolutely curbing the rise in housing prices”, real estate enterprises were obviously cautious in purchasing land, and the volume of transactions showed a clear downward trend.

### 2.2. Residential land supply prices show an upward trend

**Fig 3:**
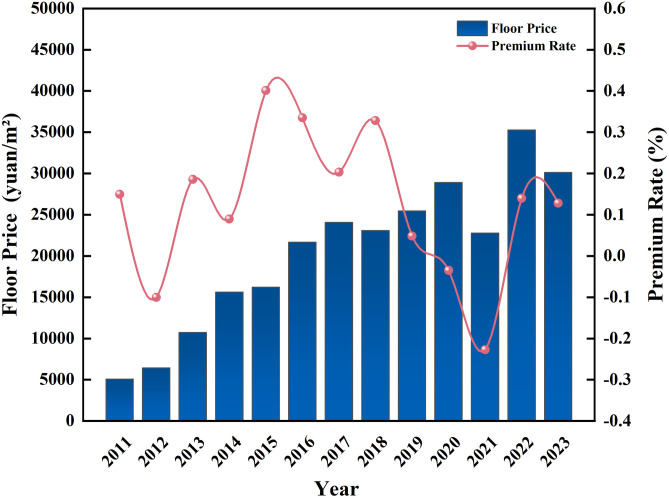
Transaction floor land price and premium rate of residential land in Beijing from 2011–2023. Data source: Wind database.

The floor land price takes into account the plot ratio, and is a better reflection of the level of land price than the land unit price. As can be seen from Fig 3, from 2011 to 2023, the floor land price has repeatedly reached record highs, rising from RMB 5,083.94 per square meter in 2011 to RMB 30,120.94 per square meter in 2023, 5.92 times increase compared to 2011. After 2016, the annual growth has been relatively flat, and the land premium rate has decreased substantially. However, in 2022, the land premium rate in Beijing experienced a significant rebound. This change is driven by multiple factors. Firstly, as the pandemic gradually came under control, the pace of economic recovery accelerated, significantly boosting developers’ confidence and investment willingness. Secondly, the Beijing municipal government adjusted its land supply strategy by increasing the availability of high-quality plots, which attracted more active participation from developers.

### 2.3. Residential land supply is gradually diversifying

**Fig 4:**
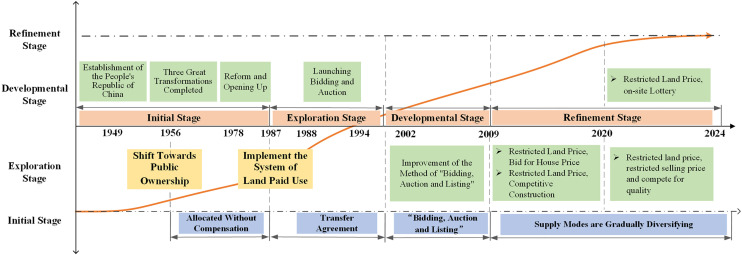
Diagram of the trajectory of urban residential land transfer policies. Note: This picture was drawn by the author.

China’s land transfer patterns has been developed mainly since 1982, and has gone through three main phases. As shown in Fig 4,the first stage is the exploratory stage (1982–2001), where the land use system was transformed from uncompensated to compensated use, and the land transfer patterns in this stage was mainly based on the agreement transfer [29]; The second stage is the development stage (2002–2008), this stage is mainly from the agreement to the “Bidding, Auction and Listing” transfer mode of change; the third stage is the improvement stage (2009 to the present). Since 2009, apart from improving the system of “Bidding, Auction and Listing”, the Beijing Municipal Government has also gradually explored new types of land disposal methods. For example, there are various forms of land transfer patterns, such as “Restricted Land Price, Competitive Construction”, “Restricted House Price, Bid for Land Price”, “Restricted Land Price, on-site Lottery” and so on. The details of the land transfer patterns are shown in Table 1.

**Table 1:** The details of the land transfer patterns.

Land Transfers Patterns	Conceptualization
Bidding, Auction and Listing	The land administration authority issues an advance notice of the auction of land plots for bidders to participate in a public place on a specified date. The auction reserve price is called out by the presiding officer, and the bidders then take turns announcing their prices in accordance with the bidding gradient, with the final parcel being awarded to the highest bidder.
Restricted Land Price, Competitive Construction	With the price of the land determined, bidders will bid upwards for the proportion of affordable housing to be built on the land, and the land will ultimately be acquired by the one with the highest proportion of affordable housing to be built.
Restricted house price, bid for land price	The land to be offered for sale is restricted to a maximum house price, and then, on the basis of the restriction on house price and household type, bidders bid upwards on the basis of the principle of “the highest bidder wins”, and downwards on the price of the house.
Restricted Land Price, on-site Lottery	If the highest price in the on-site bidding exceeds the reasonable limitation of the offer price of the land parcel, then the Municipal Land Reserve and Trade Center will hold an on-site shaking of numbers for the land parcel that has been put up for sale.
Restricted Land Price, restricted selling price, and competing for quality	In the bidding, if the reasonable limit price of the largest plot of land of the national government is reached, the bidding for the area of leased residential units or the ratio of shared ownership will start, and after reaching the upper limit of the area of leased residential units or the ratio of shared ownership, the bidding for the “commitment on the selling price of houses” will start.

### 2.4. Residential land supply structure tilted toward rental housing

The structure of residential land transfers refers to the regular combination of different uses of residential land, which tend to be distributed in horizontal space within an area. The residential land use structure is also an important basis for urban planning and rational development and utilization of land by real estate developers, reflecting the combination of various land use types. In 2015, the Ministry of Housing and Construction first proposed “rent and purchase”, and this housing system came to fruition in 2017. In order to adhere to the positioning of “housing without speculation”, adhere to the combination of rental and purchase, and strengthen the two-way adjustment of supply and demand, Beijing’s residential land supply plan will be tilted towards rental housing from 2021, and the proportion of rental housing in residential land will be increased from 13% to 30%.

## 3. Data and methodology

### 3.1. Data

This paper intends to select 67 periods of data from the 1st quarter of 2008 to the 3rd quarter of 2024 to explore the impact of the price and quantity of residential land supply on house prices. The indicators are selected as shown in Table 2. Where the average sales price of commercial housing is calculated by Equation (1).

**Table 2:** Indicator selection and data sources.

Variable	Symbol	Unit	Data Source
Average sales prices of commercial housing	lrap	yuan	National Bureau of Statistics (NBS)
Commercial housing sales area	—	m2	National Bureau of Statistics (NBS)
Commercial housing Sales	—	yuan	National Bureau of Statistics (NBS)
floor land price	lslp	yuan/ m2	Wind Database
Residential land supply area	lsls	m2	Wind Database


$$Average\;sales\;price\;of\;commercial\;housing=\frac{Commercial\;property\;sales}{Commercial\;property\;sales\;area}$$ (1)

In this paper, the values of each variable are logarithmized. On the one hand, the absolute values of the economic variables could be reduced and the heteroskedasticity could be improved; On the other hand, by taking the logarithm of the economic variables and then estimating them, the coefficients of the different influences obtained are the elasticities of the coefficients with respect to the explanatory variables.

### 3.2. Vector Autoregressive Model (VAR)

In what follows we will work with the Vector Autoregressive Model (VAR) of the following form[30]. Compared with traditional regression models, VAR models have the following three main advantages: (1) Consider the dynamic interaction between variables: it does not presuppose a strict causal structure, but rather treats all endogenous variables as a system that influences each other and evolves together, which is more in line with the real interaction between the land market and the real estate market, and helps to describe the dynamic feedback effects between prices, quantities, and housing prices. (2) Capturing lag effects and impulse responses: Adjustments in the real estate market often exhibit strong lag characteristics. VAR models can systematically capture these lag effects through the setting of lag terms and clearly demonstrate the impact path of a single variable on other variables in the system over time through impulse response functions, which is difficult to achieve with traditional static regression models. (3) Able to characterize the overall coordination and transmission mechanisms between multiple variables: VAR models are suitable for simultaneously handling the interactive effects between multiple variables and can further analyze the relative contributions of each variable to housing price fluctuations through variance decomposition, which helps to deeply understand the dynamic process of how land supply prices and quantities jointly affect housing prices.


$$Y_{t}=C+\sum_{t=1}^{p}A_{t}Y_{t-p}+\sum_{t=1}^{p}B_{t}X_{t-p}+u_{t}$$ (2)

where $Y_{t}$ is a *k*-dimensional vector of columns of endogenous variables (the quantity of land supply and the price of land supply); $X_{t}$ is a *d*-dimensional vector of columns of exogenous variables (housing price); *p* is the lag order; $u_{t}$ is the error term. $A_{t}$ and $\;B_{t}$ are the matrix of coefficients to be estimated.

### 3.3. Four-quadrant model

**Fig 5:**
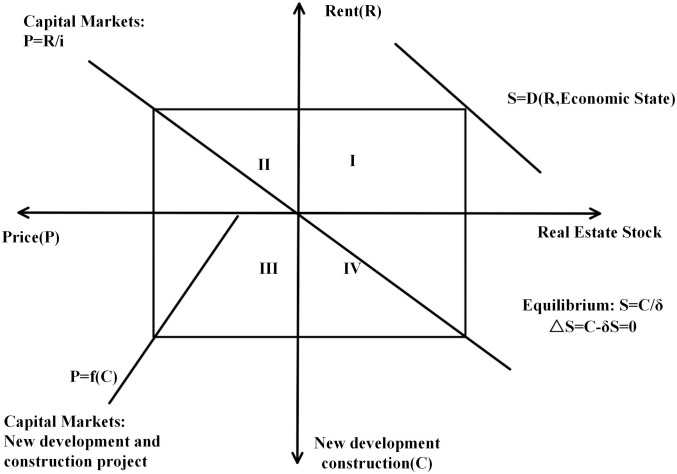
Four-quadrant model diagram.

The four-quadrant model of the real estate market was first proposed by American scholars Dipasquale and Wheaton [31]. It is used to statically analyze the operation regulations of the real estate market. It also shows the impact of the country’s macroeconomic development on the equilibrium of supply and demand in the real estate market. The model assumed that the impact of a change in a single factor on the real estate market is analyzed while other market factors remain unchanged, dividing the real estate market into the real estate using market and the real estate capital market. In Fig 5, the first quadrant represents the existing real estate stock (space market). It indicates that the existing real estate stock depends on the level of rents in a given economic situation. The second quadrant represents the real estate capitalization market. Its slope represents the capitalization rate, which indicates the expected rate of return that an investor receives from holding real estate. The third quadrant illustrates the formation of new assets in real estate. If the developer wants to make a profit, it has to develop at the equilibrium point where the price of real estate is equal to the cost of development. The fourth quadrant describes how newly developed real estate is converted to real estate stock in the real estate using market. where δ denotes the depreciation rate, and the market reaches equilibrium only when the construction of new development equals the depreciation (S = C/δ or ∆S = C-δ*S).

Although the four-quadrant model does not fully fit the Chinese context, its analytical framework still provides an analytical tool for interpreting the operation of China’s housing market. This model was proposed by American scholars Denise Dipasquale and William C. Wheaton based on the context of Western real estate markets. While Western real estate markets do differ from China’s in terms of institutional arrangements, land supply models, and market participants, the core idea of the original model is to analyze the relationships between these different markets to understand the relationship between real estate prices and rents, as well as how they are influenced by market supply and demand. Precisely because its analytical logic is relatively universal, it remains an effective tool for explaining the operational characteristics of China’s real estate market. Based on the housing market differences between China and the United States, this study improves upon the four-quadrant model referenced in Yan (2006) to explore the impact of residential land supply methods in China on the housing market [32]. In order to make the analysis of the four-quadrant model of the relationship between land transfer policy and the housing market clearer and more rational, based on the Chinese market context the following four assumptions were made:

#### Hypothesis 1.

In the primary land market, the supply of residential land is essentially an institutional allocation, which is mainly arranged by the Government through planning, policies and annual land supply plans.

#### Hypothesis 2.

In the housing market, it is assumed that the price of housing is affected only by supply and demand and is not affected by other outside factors.

#### Hypothesis 3.

The supply of residential housing is considered only for commercial and affordable housing, excluding villas and other types of housing.

#### Hypothesis 4.

In the affordable housing market, changes in supply and demand do not cause price changes in affordable housing.

### 3.4. Housing filtering model

The theory of housing filtration was originally proposed by the American sociologist Burgess when he studied the layout of housing in Chicago, revealing the underlying regularity of housing market development [33]. After the 1970s, the theory was refined by Sweeney (1974), Braid (1981) and others [34,35]. The housing market is characterized by the deterioration over time of the housing stock initially built for higher-income households and the relative decline in the price of this segment of the housing stock as the supply of new housing increases. Higher-income households abandon their existing housing in search of better living conditions, while lower-income households buy or rent such housing and continue to use it. This process is known as “housing filtering”[36]. The housing filtering model examines the quantity of new and old residences as a whole, and estimates supply and demand from the linkage between new and old residences. Therefore, the housing filtering model can be used to analyze China’s current real estate policies and their impact on the residential market.

## 4. Result

### 4.1. Impact of the price and quantity of residential land transfers on the housing market

#### 4.1.1. Model construction.

In building a VAR model to research the impact of the quantity of land supply and supply price on the price of residential housing in Beijing, the data of the selected variables must all be stable. Therefore, the augmented Dickey-Fuller (ADF) test was conducted using Stata16.0 to evaluate stationarity. The test results are shown in Table 3, both lslp and lrap do not reject the original hypothesis at the 5% significance level, indicating that the data are not stable.

**Table 3:** Unit root test for the original data.

Variables	T-statistic	5% Critical Value	P-value	Results
lsls	−5.020	−2.917	0.0000	Stable
lslp	−3.084	−2.917	0.0278	Stable
lrap	−2.039	−2.917	0.2698	Unstable

Note: * p < 0.1, ** p < 0.05, *** p < 0.01, subsequent tables are the same.

In case the original data of the variables are not stable, the variables can be processed with first-order differencing and tested for stability. The results are shown in Table 4, which shows that the significance level of the variables after first-order differencing are all less than 5%. It shows that the variables after differencing are all stable. In the following analysis we use the data after first-order differencing to build the VAR model.

**Table 4:** Unit root test for variables after first order differencing.

Variables	T-statistic	5% Critical Value	P-value	Results
dlsls	−14.740	−2.917	0.000	Stable
dlslp	−19.689	−2.917	0.000	Stable
dlrap	−11.096	−2.917	0.000	Stable

When building a VAR model, the lag order of the variables should be determined. If the lag order is chosen too small autocorrelation may occur. The larger the lag order is, the more it can reflect the dynamic characteristics of the variables in the model. However, if the lag order is too large, there will be a loss of degrees of freedom, which will affect the results of the model. Therefore, the optimal lag order is to be determined based on information criteria such as maximum likelihood, LR, AIC, BIC and HQ.

As shown in Tables 5 and 6, the final lag order selected for the residential land supply price to the residential price model is the 1st order lag, and the lag order for the residential land supply quantity to the residential price model is the 3rd order lag.

**Table 5:** Results of the lagged order test of residential land supply prices on residential price models.

lag	LL	LR	FPE	AIC	HQIC	SBIC
0	−9.901		0.005032	0.383903	0.410844	0.45252
1	7.26205	34.326*	0.003292*	−0.040711*	0.040111*	0.16514*
2	10.6911	6.8582	0.003355	−0.022295	0.112409	0.320791
3	14.0739	6.7655	0.003427	−0.002384	0.186202	0.477937
4	17.1865	6.2252	0.003534	0.026242	0.268709	0.643797

Note: * denotes the optimal lag order according to the information criterion

**Table 6:** Results of the lag order test of the quantity of residential supply on the residential price model.

lag	LL	LR	FPE	AIC	HQIC	SBIC
0	−46.2648		.016264	1.55693	1.58387	1.62554
1	−38.3604	15.809	.014341	1.43098	1.5118	1.63683
2	−29.4512	17.818	.002564*	1.27262	1.33503*	1.61571*
3	−21.5398	15.823*	.010809*	1.14645*	1.45567	1.83076
4	−19.6092	3.8612	.011581	1.2132	3.27636	4.27133

Note: * denotes the optimal lag order according to the information criterion

**Fig 6:**
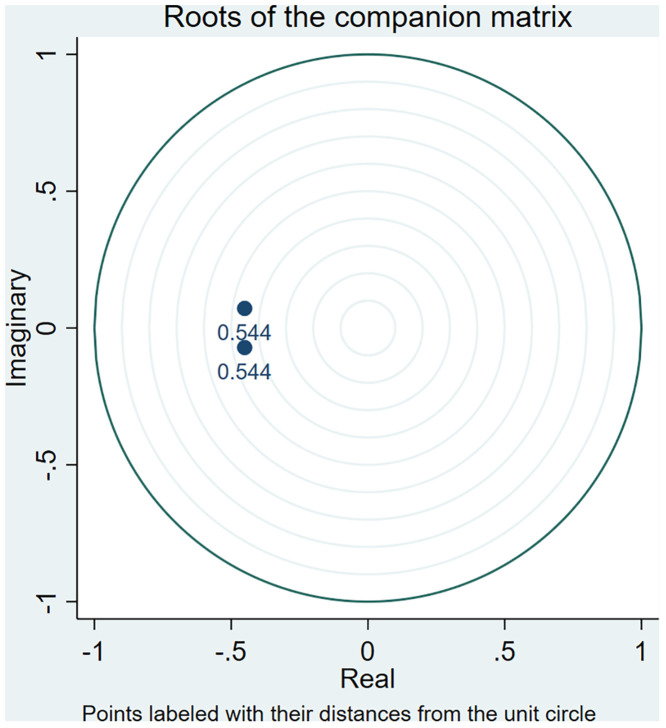
Plot of model stability test results of residential land supply price on residential price model.

**Fig 7:**
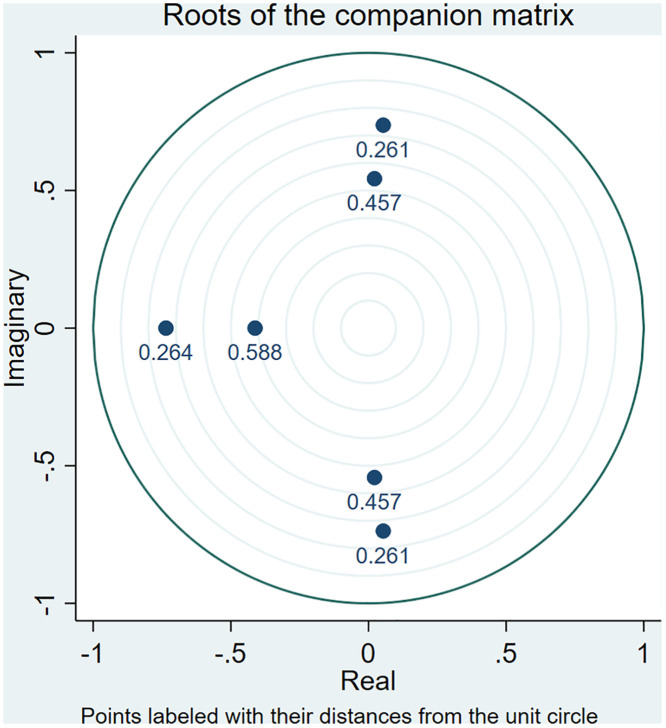
Plot of model stability test results of residential land supply quantity on residential price model.

By testing the characteristic roots of the established VAR model to determine whether the established model is reasonable. When the characteristic roots of the model are all less than 1, the estimation of the VAR model satisfies the condition of stability, which means that the VAR model established in this paper is reasonable. Fig 6 shows the test of residential land supply price on residential price model, and Fig 7 shows the test of residential land supply quantity on residential price model.

#### 4.1.2. Granger causality test.

Granger causality test is commonly used to identify the causal relationship between variables. This test is an association test rather than a strict causal inference. It is used to judge the predictive ability of past variables on current variables and cannot prove the true causal mechanism. In this paper, Granger causality test was conducted by using Stata 16.0 software to explore the relationship between land supply price and land supply quantity on residential sales price and the results are shown in Table 7.

**Table 7:** Granger causality test results table.

Equation	Excluded	chi2	df	P-value
dlrap	dlslp	5.227 5	1	0.025 7
	ALL	5.227 5	1	0.025 7
dlslp	dlrap	0.937 43	1	0.336 7
	ALL	0.937 43	1	0.336 7
dlsls	dlrap	3.491 5	3	0.021 4
	ALL	3.491 5	3	0.021 4
dlrap	dlsls	0.124 39	3	0.945 3
	ALL	0.124 39	3	0.945 3

The results in Table 7 indicate that at the 5% significance level, the floor land price (dlslp) is the cause of the average sales price of commercial housing (dlrap); the residential land supply area (dlsls) is the cause of the average sales price of commercial housing (dlrap); and the average sales price of commercial housing (dlrap) is not the cause of the floor land price (dlslp), but the residential land supply area (dlsls). Therefore, it can be assumed that changes in land prices will have an impact on Beijing’s housing prices, while the change in housing prices can influence the supply of land.

Similarly, other scholars have also centered their research on the relationship between land prices or the quantity of land supply and house prices. The difference is that Grimes (2010) focuses on the relationship between land and house prices in the New Zealand region from the micro perspective of land supply elasticity and found that rising land prices weaken the housing supply elasticity and exacerbate the volatility of house prices, and argues that flexible land supply mechanisms can help to mitigate the trend of house price rises [37]; whereas Sun and Dong (2017) focuses on the relationship between macro policies and the urbanization development to analyze the impact between land market and house prices [38]. The results found that in mega-cities such as Beijing, Shanghai and Shenzhen, the inhibitory effect of increased land supply on house prices is stronger, while in some small and medium-sized cities in China, the effect of increased land supply on house prices is not significant. Likewise, Hilber and Vermeulen (2016) find that land supply constraints in the UK affect house prices through the triple channel of regulatory constraints, land scarcity, and topographical constraints, with regulatory constraints being the most important driver of house prices, and their effect is more pronounced in areas with highly restricted land supply such as London, as well as in areas with high levels of urbanization and economic prosperity[39]. By comparing the results with those of this paper, we find that the land supply system affects the operation of the housing market through similar mechanisms, both in mega-cities such as Beijing Shanghai and Shenzhen in China, and in large cities in different institutional environments such as New Zealand in foreign countries. Such a comparative analysis further enhances the robustness, generalizability and theoretical support of this paper’s findings.

#### 4.1.3. Impulse response.

(1) **Impulse Responses to House Price Fluctuations Induced by Residential Land Supply Price Shocks**

**Fig 8:**
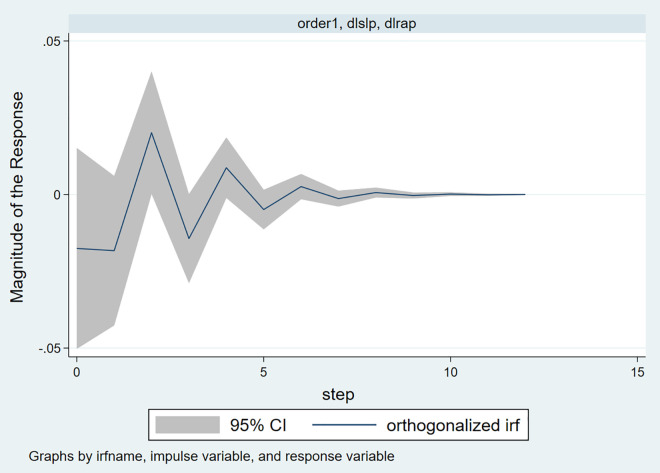
Impulse responses to house price fluctuations induced by residential land supply price shocks.

Fig 8 shows the impulse response of housing price fluctuations due to land supply price shocks. From the Fig 8, it can be observed that when land supply prices rise, housing prices exhibit a negative reaction in the current period, followed by a rapid rebound, housing prices begin to show a significant positive fluctuation, which gradually weakens and stabilizes. This dynamic process reflects the complex time-lagged transmission relationship between the land market and the housing market.

Specifically, an increase in land supply prices means higher land acquisition costs for real estate developers. Due to the time lag in the development cycle, the increase in land prices does not immediately transmit to housing prices in the short term. Instead, developers often face compressed profit margins in the early stages of rising land costs, and may hesitate to raise prices rapidly due to unclear market confidence. Therefore, in the period of the shock, housing prices respond with a slight decrease or a delayed adjustment due to “price rigidity,” forming a short-term negative impact. In the second period, as new projects with high land prices gradually enter the market, real estate companies begin to pass on the cost pressures to downstream homebuyers through price adjustment strategies. The increase in land prices is gradually reflected in the future sale prices of projects, driving housing prices to experience positive fluctuations. This transmission reflects the “land price-housing price linkage” mechanism. In particular, in markets with a limited land supply structure, urban land scarcity, and little competition, developers have more bargaining power, which accelerates the rise in housing prices. During the second to fourth periods of housing price increases, the expectations of market participants also play a role in amplifying the effect. The rise in land prices is often seen as a policy signal from the government regarding “land supply tightening” or “housing price support.” Homebuyers expect that housing prices will continue to rise in the future, which may accelerate their purchasing pace, further stimulating demand and, in turn, driving housing prices up. This feedback mechanism reflects the herd effect driven by signals in the real estate market. Starting from the fifth period, the housing price response gradually stabilizes. This stabilization is likely driven by two factors. First, as the shock is gradually absorbed, the transmission chain between land prices and housing prices completes, and the marginal effect diminishes; Second, the government often implements related regulatory policies (such as price limits, purchase restrictions, and increased land supply) to curb the rapid rise in housing prices, strengthening market expectation management. These interventions help achieve policy goals of controlling housing price fluctuations and stabilizing the market. Therefore, in the long run, both policy regulation mechanisms and the rebalancing of supply and demand in the market jointly contribute to the stabilization of price responses.

(2) **Impulse Response to House Price Fluctuations Induced by Shocks to Quantity of Residential Land Supply**

**Fig 9:**
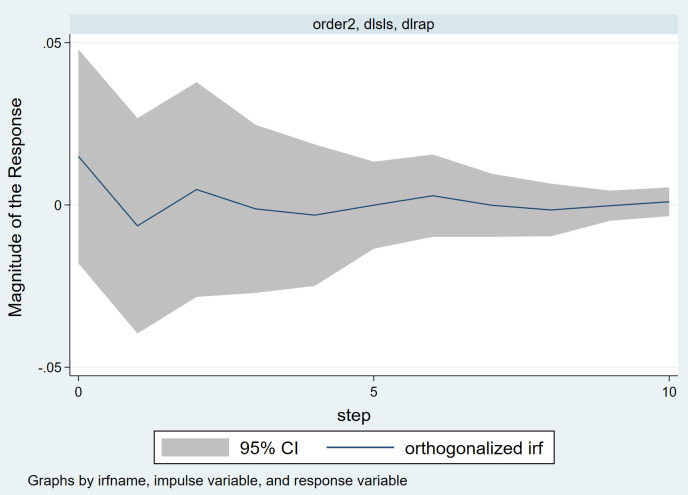
Impulse response to house price fluctuations induced by shocks to quantity of residential land supply.

Fig 9 illustrates the impulse response of housing price fluctuations due to shocks in land supply quantity. It can be observed that an initial increase in land supply volume leads to a slight decline in housing prices, reflecting the market’s preliminary reaction to the supply of new land. In the short term, the real estate market’s supply-demand relationship cannot adjust immediately, and developers, when confronted with an increase in supply, may experience pricing pressure due to expectations of an oversupplied land market. Particularly when the market has not yet fully absorbed the newly supplied land, short-term market sentiment often reflects concerns about “oversupply,” resulting in temporary downward adjustments in housing prices. As time progresses, beginning from the second period, housing prices start to exhibit a positive fluctuation, indicating a lagged effect of increased land supply on housing prices. This lag reflects a shift in market expectations regarding future housing supply. Although the supply of land has increased, the process of turning land into housing requires a considerable amount of time, and the increase does not immediately translate into effective housing supply. Consequently, while housing demand remains strong, the actual supply in the short term does not meet that demand, thereby pushing up housing prices. This reflects the time-lag effect in the real estate market. From the third period onward, the figure shows a noticeable negative impact, which gradually weakens and eventually stabilizes. This delayed negative effect suggests that as housing projects are completed and converted into effective supply, market demand begins to saturate, leading to a downward adjustment in housing prices. The gradual weakening and eventual stabilization of this negative impact can be attributed to the combined influence of government macroeconomic regulation and market expectation adjustments. The government introduce relevant policies—such as stricter regulation of land supply or restrictions on speculative demand—to effectively guide market expectations and mitigate excessive housing price volatility. At the same time, the gradual adaptation of market participants and their growing confidence in the release of future supply also play a crucial role in stabilizing housing prices.

### 4.2. Impact of the land transfer pattern on the housing market

This section is primarily based on the four-quadrant theory model, which explores the impact of different residential land transfer methods on housing market prices from a theoretical perspective and analyzes the intrinsic interactive relationship between land transfer methods, land prices, and housing prices. Through this theoretical analysis, we aim to enrich our understanding of the operating mechanisms of the land transfer market and provide a reference for subsequent policy recommendations.

#### 4.2.1. “Bidding, Auction and Listing”.

**Fig 10:**
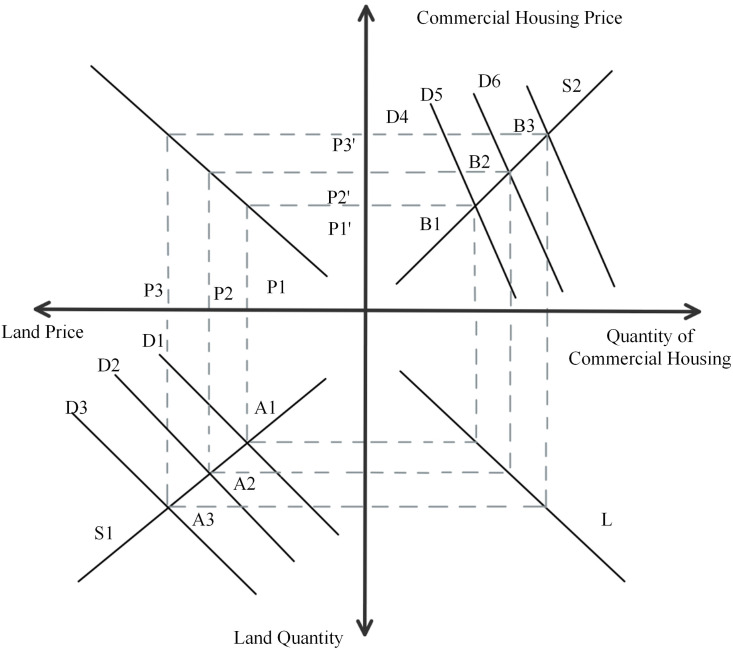
Four-quadrant model of the “Bidding, Auction and Listing” pattern.

From Fig 10, we can know that the first quadrant is the commercial housing market and the third quadrant is the land transfer market. In the land market, the demand curve intersects the supply curve at point A1. The land price level at this point is P1 and the sales price of commercial housing is P1’. The sustained rise in land prices has contributed to higher housing prices, intensifying public fears that housing prices may continue to climb. So, the demand curve from D4 to the right to D5, and S2 curve intersects at point B2, at this time the sales price of commercial housing from P1’ to P2’. The sales price of commercial properties has a tendency to rise due to the combined effect of rising land prices and rising expectations, which will release a favorable signal to developers. As a result, developers’ induced demand for land will increase and the competition among developers will become more intense, leading to rising land prices. Rising land prices will further push up house prices, eventually forming a vicious cycle of rising land prices and house prices. According to statistical data, the average sales price of commercial housing in Beijing surged from 5,243 yuan per square meter in 2002 to 16,846 yuan per square meter in 2009. This significant upward trend strongly corroborates the aforementioned conclusion.

#### 4.2.2. Restricted land price, competitive construction.

**Fig 11:**
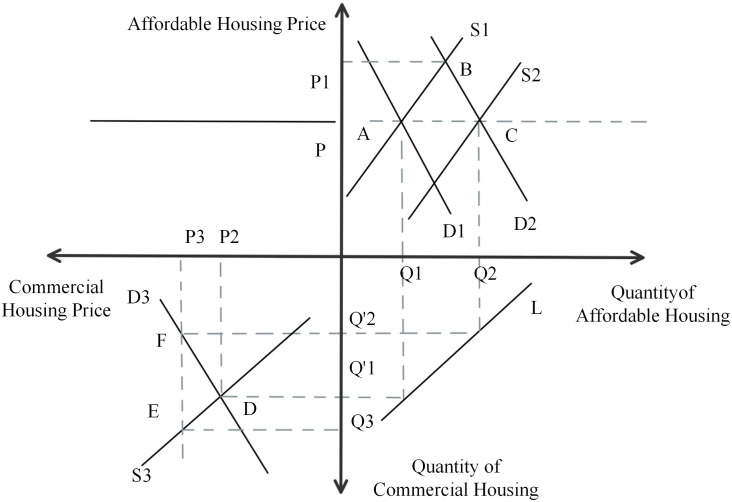
Four-quadrant model of the “Restricted Land Price, Competitive Construction” pattern.

From Fig 11, we can know that the first quadrant is the market for affordable housing and the third quadrant is the market for commercial housing. Assuming that initially the supply curve for affordable housing is S1 and the demand curve is D1. At this point, the equilibrium quantity of sheltered housing is Q1 and the price is P. The equilibrium quantity of commercial housing is Q’1 and the equilibrium price is P2. Because the price of sheltered housing will be lower than the price of commercial housing, the demand for sheltered housing will increase. The equilibrium price at this point is P1 and the equilibrium quantity is Q1. The price of government-built affordable housing is constant, so in order to maintain this price level of P, the government will increase supply and build more affordable housing, at which point the supply curve will shift from S1 to the right to S2, intersecting with the D2 curve at point C. At this point the equilibrium price decreases from P1 to P and the equilibrium quantity increases from Q1 to Q2. After acquiring land through the “Restricted Land Price, Competitive Construction” pattern, on the one hand, developers have to build commercial housing, and on the other hand, they have to build affordable housing. Since there is a limit to the total price of the land acquired, the development cost will increase if it builds a higher proportion of affordable housing. The developer can only make up for the development cost of affordable housing by building more commercial housing and raising the price of commercial housing. When the price of commercial housing rises to P3, the demand for commercial housing falls from Q’1 to Q’2, while the supply of commercial housing at this time is Q3, giving rise to a gap between supply and demand of (Q3 - Q’2).

#### 4.2.3. Restricted house price, bid for land price.

**Fig 12:**
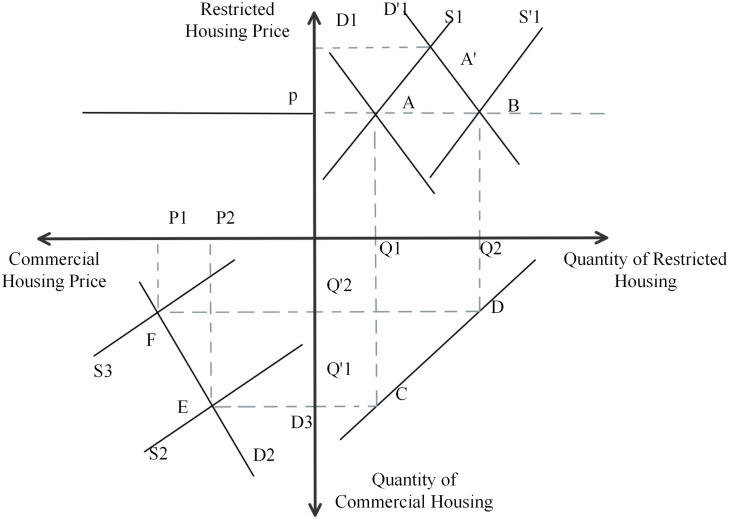
Four-quadrant model of the “Restricted House Price, Bid for Land Price” pattern.

As can be seen in Fig 12, the first quadrant is the restricted housing market and the third quadrant is the commercial housing market. Suppose that initially the supply curve is S1 and the demand curve is D1, at which point the market equilibrium is A, the equilibrium price is P, and the equilibrium quantity is Q1. Since the price of bid-restricted housing is lower than the price of commercial housing, the demand for bid-restricted housing will increase, so the demand curve shifts from D1 to the right to D’1 intersecting the S1 curve at point A’. The price will be stabilized at P because the price of restricted housing limits the maximum and average price. In order to stabilize the price at the level of P, the government will increase the supply of restricted housing, so the supply curve shifts from S1 to the right S’1 and the D’1 curve intersects at point B. At this point, the demand for restricted housing is Q2. The increase in restricted housing will lead to a decrease in the supply of commercial housing, at which point the supply curve of commercial housing moves from S2 to S3 intersecting the demand curve D2 at point F. The equilibrium price is P1 and the equilibrium quantity is Q’2. The market for commercial housing will also reach a new equilibrium, but at a higher price than before.

#### 4.2.4. Restricted land price, on-site Lottery.

**Fig 13:**
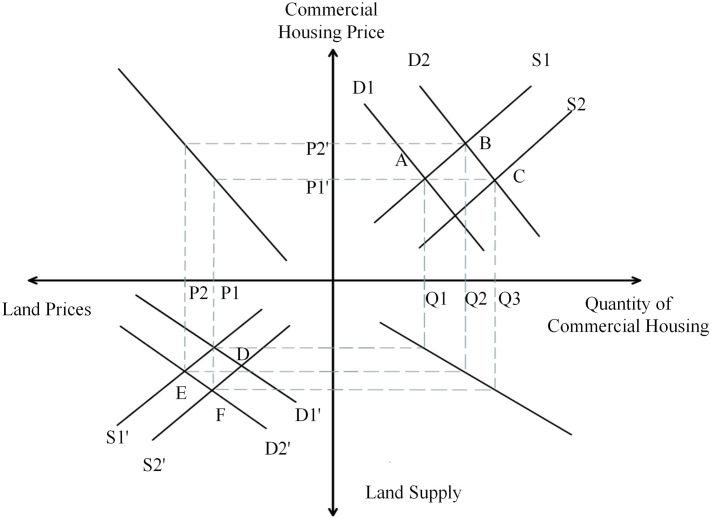
Four-quadrant model of the “Restricted Land Price, on-site Lottery” pattern.

From Fig 13, the initial land supply curve is S1’ and the demand curve is D1’, the equilibrium point is point D, and the equilibrium price is P1, the equilibrium quantity of commercial housing is Q1 and the equilibrium price is P1’. When the demand for housing increases, the demand curve shifts from D1 to D2 and intersects the supply curve S1 at point B. The equilibrium price is P2’ (P2’ > P1’) and the equilibrium quantity is Q2. As people’s demand for commercial housing increases, it will lead to an increase in the demand for land as well, at which point the demand curve will shift from D1’ to D2’, intersecting with the supply curve at point E. The equilibrium price of land will rise from P1 to P2 and the price of commercial housing will rise from P1’ to P2’. If the government increases the quantity of land supply through the means of “Restricted Land Price, on-site Lottery”, then the land supply curve will move from S1’ to S2’ and intersect with the D2’ curve at point F, and the price of land will soon return to the limiting price of P1. At this time, the commercial housing market will reach a new equilibrium at point C, and the price of commercial housing will be stabilized at P1’.

#### 4.2.5. Restricted land price, restricted selling price and compete for quality.

**Fig 14:**
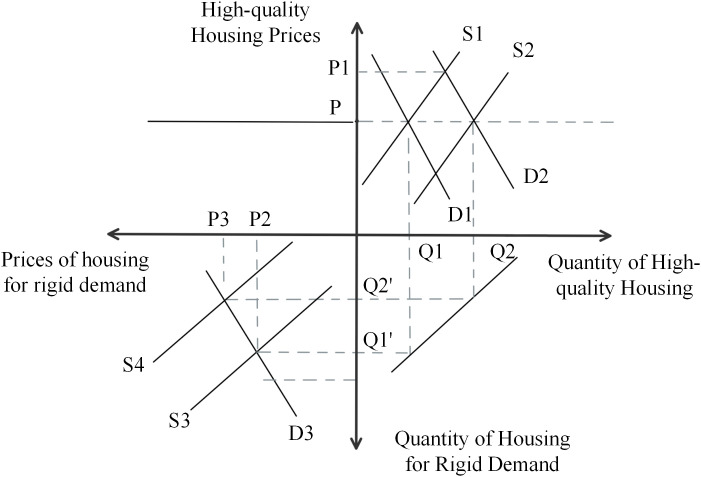
Four-quadrant model of the “Restricted Land Price, Restricted Selling Price and Compete for Quality” pattern.

As can be seen in Fig 14, the initial supply curve for high-quality housing is S1 and the demand curve is D1, at which point the equilibrium price is P. Due to the opening of the two-child and three-child policy, the size of the family structure will expand, and the demand for improved housing will increase, so the demand curve will shift from D1 to the right to D2, and at this time the equilibrium price of high-quality housing rises from P to P1. Because of the “limit land price, limit selling price, bidding for quality” policy, will control the price of housing. At this time, the government regulates the price of high-quality housing by increasing supply, and the supply curve shifts from S1 to the right to intersect with the demand curve D2, at which time the equilibrium price of high-quality housing returns to the original price level P. Due to the limited quantity of land supply, an increase in the supply of high-quality housing leads to a corresponding decrease in the supply of rigid demand housing. Then, the supply curve of rigid housing shifts from S3 to S4 intersecting with the demand curve D3, the equilibrium quantity falls from Q1’ to Q2’ and the equilibrium price rises from P2 to P3. It can be seen that the land transfer pattern of “Restricted Land Price, Restricted Selling Price and Compete for Quality” guarantees the supply of high-quality housing, but to a certain extent, it may lead to the supply of rigid demand housing being controlled, thus pushing up the price of rigid demand housing.

#### 4.2.6. Advantages and disadvantages of transfer patterns.

By analyzing the five land transfer patterns, the advantages and disadvantages of the different transfer patterns are shown in Table 8. The optimal land transfer patterns can be selected according to the local policy.

**Table 8:** Comparison of the advantages and disadvantages of the five land transfer patterns.

Transfer Pattern	Advantages	Disadvantages
“Bidding, Auction and Listing”	Introduces a market competition mechanism Providing a fair and transparent market environment Adherence to the principle of “highest bidder wins”	Repeated bidding by bidders has led to escalating land sale prices, fueling the rise in land prices
“Restricted Land Price, Competitive Construction”	Increased the construction of affordable housing, which is conducive to solving the problem of housing difficulties for low-income people	Causing an oversupply of high-grade commercial housing The co-construction of affordable and high-grade commercial housing may create neighborhood conflicts
“Restricted House Price, Bid for Land Price”	Ability to control land and house prices	Cannot be sold for 5 years after purchase With limited profit margins, developers may squeeze costs and compromise the quality of houses Restricted demand for improved housing
“Restricted Land Price, on-site Lottery”	Controlling land premium space Guaranteeing the fairness and impartiality of the land transfer market	The qualification level of the developer selected by the on-site lottery is not necessarily optimal
“Restricted Land Price, Restricted Selling Price and Compete for Quality”	Ability to control land and house prices Ability to control the maximum price of housing Pay more attention to the design of household space, living experience, and the perfection of supporting facilities	

As can be seen from Table 8, different ways of land transfer have their own advantages and limitations. The traditional “Bidding, Auction and Listing” mechanism creates an open, fair and just market environment through the introduction of market competition, effectively curbing the occurrence of rent-seeking behavior. The principle of “the highest bidder wins” also helps to attract the participation of real estate enterprises with strong capital strength and rich development experience, which is conducive to improving the overall quality and level of residential construction. However, against the background of scarce land resources and fierce competition, frequent bidding has pushed up land prices, resulting in high land transfer prices, which in turn has pushed up the price of commercial housing. In order to address the problem of rapidly rising land prices under the “Bidding, Auction and Listing” mode, the “Restricted Land Price, Competitive Construction” mode has emerged. By setting a ceiling on land prices and guiding developers to compete in the affordable housing, this mode stabilizes land prices while helping to alleviate the housing pressure on low- and middle-income groups. The mode of “Restricted House Price, Bid for Land Price” tries to curb the rise of house price by controlling the future sales price, but since the house price is locked at the stage of land disposal, developers, with limited profit margins, tend to control the expenditure by compression of cost, which may bring about the hidden danger of the decline of the quality of the houses. The “Restricted Land Price, on-site Lottery” mode to a certain extent to avoid vicious competition, improve the fairness of the land transfer process, but there is also the problem of uneven qualifications of the successful developers, the quality of the implementation of the project is difficult to guarantee. Based on the experience of the above transfer mode, the mode of “limiting land price, limiting house price and competing for quality” has been gradually promoted. This pattern not only controls the premium level of land price and house price, but also guides developers to compete for quality in design, construction and supporting facilities, effectively realizing the dual goals of price control and quality assurance, and helping to promote the sustainable development of the real estate market.

By contrast, the land transfer policies of other countries are quite different from those of China. Singapore, for example, has adopted a dual land supply mechanism of “government & market”. After compulsory acquisition and consolidation of land, the government supplies land to the market by means of “sealed bid auction”, reflecting a strong attribute of planning and control; while private land is mainly traded through “collective sale”. Ooi et al. (2011) pointed out that the government regulates the scale of land supply through sealed bid auction, which can effectively curb overheating in the market and prevent excessive rise in land prices, thus stabilizing the expectation of house prices to a certain extent [40]. In contrast, the United States land market is based entirely on a system of private property rights. Federal and local governments are usually not directly involved in the sale of land, but rather influence development behavior through the Planning Permission system. Gyourko and Molloy (2015) found that in highly regulated areas such as Boston and San Francisco, natural geography and policy constraints form a “double constraint” on land supply, making it difficult to quickly release new land supply, thus exacerbating the contradiction between supply and demand in the market, and pushing up local house price increases[41].

### 4.3 Impact of the transfer structure on the housing market

The paper divides the housing market into “four markets” filtering model to study the impact of the different proportion of land transfer structure on the real estate market. It is assumed that the first-class market is the rental housing market; the second-class market is the second-hand housing; the third-class market is the general commercial housing market; and the fourth-class market is the high -grade commercial housing market. The rental housing market primarily focuses on guaranteed rental housing. Guaranteed rental housing is government-supported land, fiscal, tax, financial and other policies, giving full play to the role of the market, and is rented out as guaranteed housing for those who do not have a home in urban areas, especially groups such as those engaged in basic public services, mainly to solve their housing difficulties.

**Fig 15:**
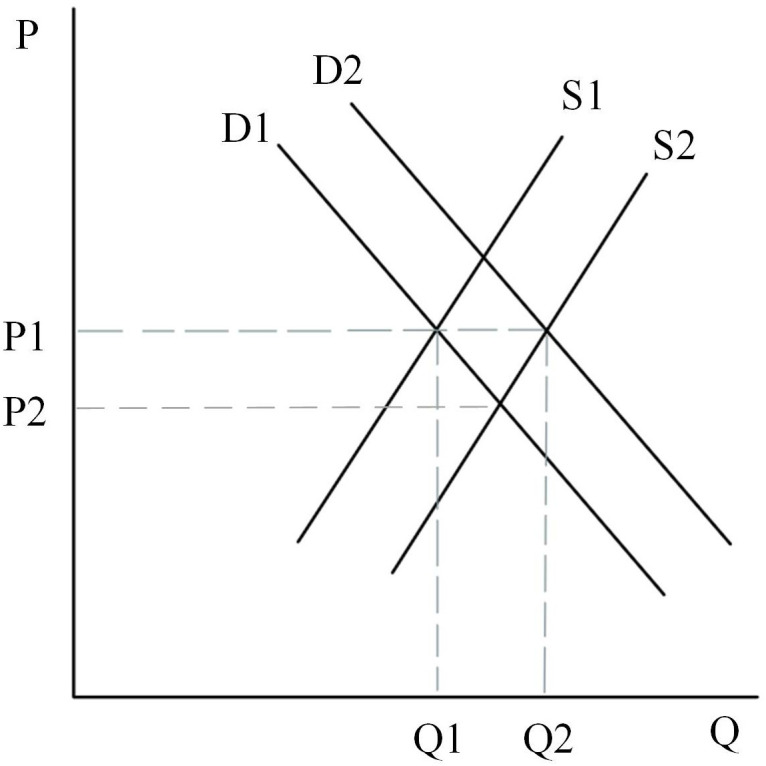
Rental housing market.

Firstly, the initial rental market is assumed to be determined by D1 and S1 in Fig 15, and at this point the equilibrium price in the rental market is P2 and the equilibrium quantity is Q1. As the land supply policy is tilted toward land for rental housing, the land for rental housing will gradually increase, and the supply curve will shift from S1 to the right to S2 intersecting with the D1 curve, at which time the equilibrium rental price will fall from P2 to P1. However, the reduction of land for ownership housing will make some consumers filter from the general commercial housing market to the rental market, thus making the demand for rental housing increase, at which time the demand curve will shift from D1 to the right to D2 intersecting with the supply curve S2. At this point, the price of the rent reverts to the original P2.

**Fig 16:**
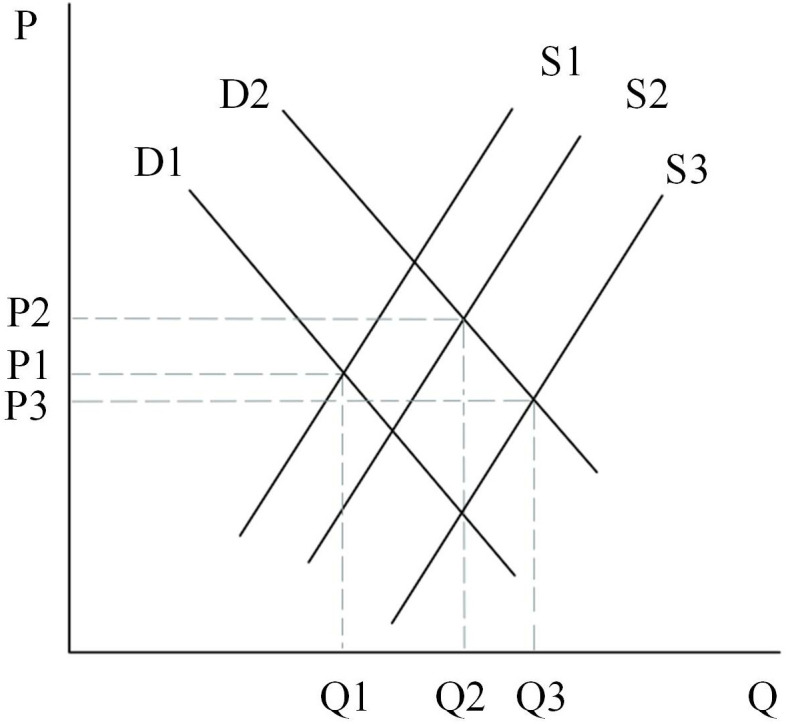
General commercial housing market.

Secondly, the initial equilibrium state of the second-hand housing market is represented by the point where the D1 and S1 curves intersect in Fig 16. As the supply of ordinary and high-grade commercial housing decreases, some households with lower incomes that cannot afford high-grade and ordinary commercial housing can only filter down to the second-hand housing market or temporarily choose rental housing, at which point the demand for second-hand housing increases, and the demand curve shifts from D1 to D2 to the right. The supply in the second-hand housing market will increase as a portion of high-grade housing filters into the second-hand housing market. The equilibrium price and equilibrium quantity in the market are determined by supply and demand, and at this point it is necessary to consider the situation in two ways. First case: when the supply curve moves from S1 to S2 intersecting D2. At this point, high-grade housing filters down to the second-hand market, making the number of new second-hand houses less than the quantity demanded, which can make the second-hand market oversupplied, leading to price increases, which in turn triggers the phenomenon of second-hand housing inversion; Second case: when the number of second-hand house supply exceeds the demand, the quantity of supply in the second-hand house market increases and the price decreases.

**Fig 17:**
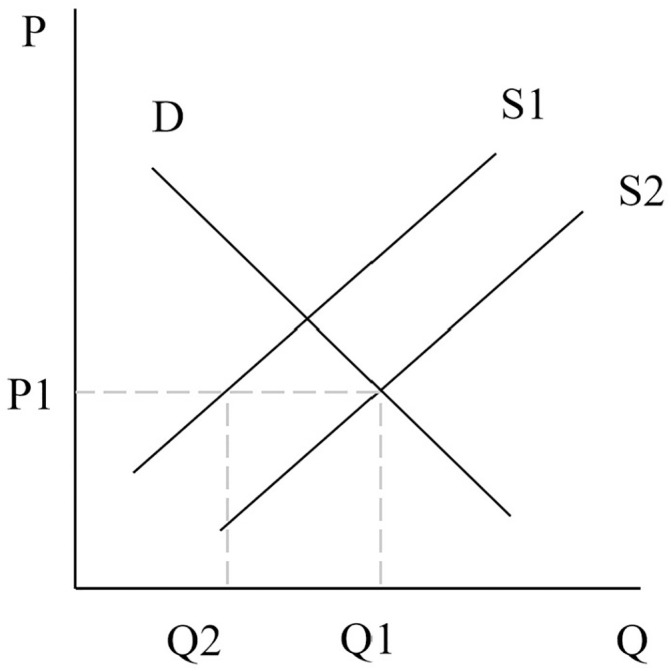
General commercial housing market.

Thirdly, Fig 17 represents the general commercial housing market, assuming that the initial equilibrium state of the market is determined by the demand curve D and the supply curve S1. At this point the equilibrium quantity is Q1 and the equilibrium price is P1. As the proportion of land supply in the ownership category decreases, the supply of general commercial housing will also decrease, so the supply curve shifts from S1 to S2 and intersects with the demand curve D. Because of the strategy of limiting the price of ordinary housing, the price of housing will be maintained at the level of P1, at this time, there will be an oversupply of demand, which will produce a gap between the supply and demand of (Q1-Q2).

**Fig 18:**
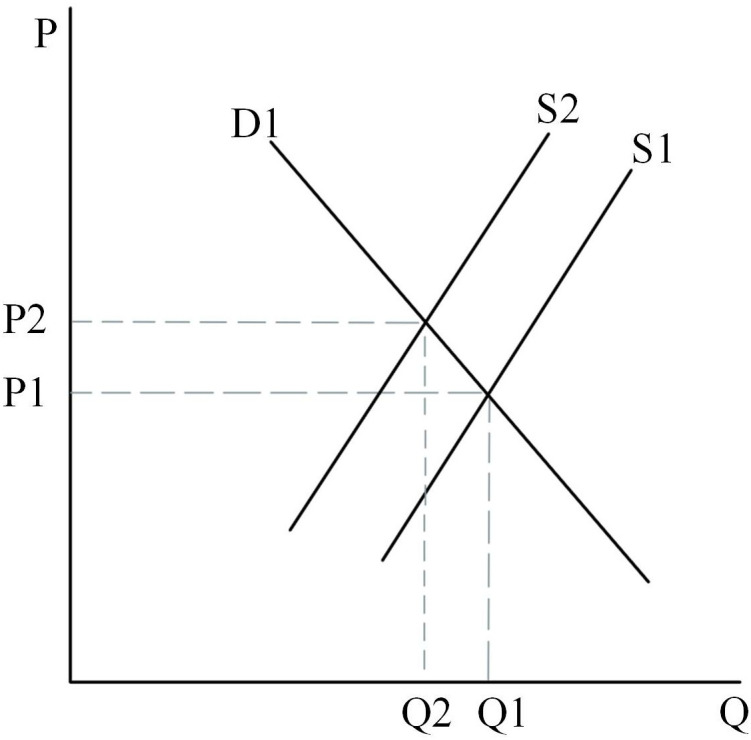
High-grade commercial housing market.

Finally, assume that the initial market for high-grade housing is determined by D and S1 in Fig 18, at which point the equilibrium quantity in the market is Q1 and the equilibrium price is P1. As the land supply policy is tilted towards rental residential land, the quantity of land supply for high-grade commercial housing will be reduced, which leads to a shift in the supply curve of high-grade commercial housing from S1 to S2 intersecting with D. At this time, the equilibrium price rises from P1 to P2, and the equilibrium quantity falls from Q1 to Q2. Therefore, the main effects brought about in the high-grade commercial housing market are an increase in the price of commercial housing and a decrease in the volume of transactions.

Similar to this study, other scholars have conducted extensive research around the relationship between the supply of land for affordable housing and house prices. Chen and Wu (2021), Han and Feng (2024) argued that mega-cities such as Beijing, Shanghai, Shenzhen and others will lead to a vicious circle in which “the more affordable housing is built, the less commercial housing is supplied, and the higher the price of commercial housing is”, due to the constraints of rigidity of land and the differentiation of demand structure [42,43]. Stacy et al. (2023) find that in high-priced cities with limited land resources, such as Los Angeles, an increase in the supply of land for affordable housing may squeeze the supply of land for commercial housing, leading to an increase in the price of land bid by developers and indirectly pushing up the price of housing[44]. The results of these studies in different contexts are the same as those in this paper, suggesting that in the context of high-priced cities with tight land supply, the policy of supplying affordable housing may squeeze the supply of the commercial housing market, thus pushing up the price of commercial housing.

## 5. Conclusions

The study systematically analyzes the impact mechanism of land transfer systems on the housing market, taking Beijing as a representative case. It reveals the effects of policy regulation across four dimensions: land supply quantity, structure, mode, and price. This finding enriches the understanding of the price impact mechanism of the housing market in the context of land system reforms.

Firstly, land supply prices have a significant positive impact on housing prices. The higher the land price, the more likely housing prices will rise. However, an increase in land supply quantity has a delayed negative impact.

Secondly, in terms of the land transfer mode, Beijing’s “Bidding, Auction, and Listing” system with high premium rates and rising land prices has created a market competition pattern similar to other first-tier mega-cities. This suggests that the model generally exacerbates upward pressure on housing prices. However, this study further reveals that Beijing’s new land transfer mechanisms, such as “Restricted Land Price, on-site Lottery” and “ Restricted Land Price, Restricted Selling Price, and Competing for Quality” demonstrate strong institutional flexibility in controlling land prices and stabilizing housing prices. This provides valuable lessons for other cities exploring differentiated land transfer policies.

Thirdly, models such as “ Restricted Land Price, on-site Lottery “ and “ Restricted Land Price, Restricted Selling Price, and Competing for Quality” help to reduce irrational competition among developers and can, to some extent, stabilize market expectations and ensure the quality of housing. This type of land transfer model holds significant reference value for regions implementing government-led land supply systems. By strengthening comprehensive regulation over land use, transfer prices, and housing construction quality, it helps alleviate excessive fluctuations in the real estate market and promotes the stable and healthy development of the housing market.

Additionally, regarding the impact of rental land on market structure, this study finds that changes in the supply structure of rental land have a significant interlinking effect on the supply and demand of housing and rental prices. Low-and middle-income households, due to the lack of a stable rental assistance system, are more likely to turn to the second-hand housing market due to supply-demand mismatches, which indirectly drives up second-hand housing prices and rental levels. Thus, through long-term rental contracts, government subsidies, and housing guarantees, such structural supply-demand imbalances can be alleviated to some extent.

Based on the above findings, the policy recommendations given in this paper are as follows:

First, real estate market regulation should adhere to the principle of dual-directional adjustment. During periods of rapid housing price increases, expanding land supply and controlling land transfer prices are conducive to stabilizing housing prices and promoting the healthy and sustainable development of the residential market. Conversely, in periods of declining housing prices, reducing land supply can help avoid excessive market downturns and prevent a “housing price–land price” downward spiral that could pose fiscal and economic risks. Cities of different types should implement elastic adjustments when applying the dual-directional approach. For example, first-tier cities, which experience strong net population inflows and high housing demand, should place greater emphasis on supply-side regulation, whereas smaller cities should guard against oversupply that may result in high vacancy rates and fiscal stress.

Second, differentiated land supply strategies should be adopted based on the spatial characteristics of each city. Governments should formulate land release conditions tailored to the functional attributes and population structure of different areas, thereby enabling more precise regulation. For high-quality housing in central urban areas, the conventional “Bidding, Auction, and Listing” mechanism can remain dominant. In contrast, for housing that meets rigid demand, priority should be given to combined mechanisms such as “Restricted Land Price, Competitive Construction” and “Restricted House Price, Bid for Land Price,” to balance market efficiency with social welfare. In suburban or newly developed areas, mechanisms like “Restricted Land Price, Restricted Selling Price, and Competing for Quality” can be explored to encourage developers to improve housing quality while keeping prices affordable.

Third, a more flexible land supply policy should be adopted. Major cities generally face significant land constraints, especially megacities, where such pressures not only drive up housing prices but also worsen affordability issues for low- and middle-income groups. It is therefore essential to enhance the coordination between the “rent-and-buy” housing system and medium- to long-term land supply forecasting and regulation mechanisms, and to establish a dynamic land supply model. Specifically, (1) the proportion of land allocated for rental housing should be arranged scientifically to meet the needs of the “sandwich class” through the construction of policy-based rental housing, thus preventing excessive rent hikes; (2) the share of land for improvement-oriented housing should be appropriately increased to meet the quality living demands of middle- and high-income households and avoid their crowding out of ordinary housing resources by turning to high-end markets.

##  6. Discussion

Although this paper uses VAR models and Granger causality tests to analyze the dynamic relationship between land supply quantity, supply price, and housing prices, and reveals the time series correlation and predictive relationship between them, it should be emphasized that these empirical results cannot be regarded as strict causal inferences. Firstly, Granger causality tests are mainly used to test the predictive power between variables, i.e., whether the past information of one variable can significantly improve the prediction of another variable, and does not represent a strict causal relationship. Secondly, there are complex two-way feedback mechanisms in the real estate market. For example, rising housing prices may influence local governments’ land supply decisions, forming an endogenous feedback loop that is difficult to completely isolate within the VAR framework. Therefore, the empirical findings of this paper are better understood as dynamic correlations and predictive relationships between variables rather than definitive causal relationships. Future research could employ traditional regression models combined with more exogenous shocks or instrumental variable methods to achieve a more rigorous causal identification of the impact of land supply policies on housing prices.

## Supporting information

S1 Data Data for plotting **Fig 1** **.**
(XLSX)

S2 Data Data for plotting **Fig 2** **.**
(XLSX)

S3 Data Data for plotting **Fig 3** **.**
(XLSX)
